# Technical Aspects of Flow Cytometry-based Measurable Residual Disease Quantification in Acute Myeloid Leukemia: Experience of the European LeukemiaNet MRD Working Party

**DOI:** 10.1097/HS9.0000000000000676

**Published:** 2021-12-22

**Authors:** Jesse M. Tettero, Sylvie Freeman, Veit Buecklein, Adriano Venditti, Luca Maurillo, Wolfgang Kern, Roland B. Walter, Brent L. Wood, Christophe Roumier, Jan Philippé, Barbara Denys, Jeffrey L. Jorgensen, Marie C. Bene, Francis Lacombe, Adriana Plesa, Monica L. Guzman, Agnieszka Wierzbowska, Anna Czyz, Lok Lam Ngai, Adrian Schwarzer, Costa Bachas, Jacqueline Cloos, Marion Subklewe, Michaela Fuering-Buske, Francesco Buccisano

**Affiliations:** 1 Department of Hematology, Amsterdam UMC, Location VUmc, Amsterdam, The Netherlands; 2 Department of Clinical Immunology, Institute of Immunology and Immunotherapy, College of Medical and Dental Sciences, University of Birmingham, United Kingdom; 3 Department of Medicine III, University Hospital, LMU Munich, Germany; 4 Department of Biomedicine and Prevention, University of Rome Tor Vergata, Rome, Italy; 5 MLL Munich Leukemia Laboratory, Munich, Germany; 6 Clinical Research Division, Fred Hutchinson Cancer Research Center, Seattle, Washington, USA; 7 Department of Pathology and Laboratory Medicine, Childrens Hospital Los Angeles, California, USA; 8 Laboratory of Hematology, CHU Lille, France; 9 Department of Laboratory Medicine, Ghent University Hospital, Ghent, Belgium; 10 Department of Hematopathology, UT MD Anderson Cancer Center, Houston, Texas, USA; 11 Hematology Biology, Nantes University Hospital, Nantes, France; 12 Flow Cytometry Platform, University Hospital, Bordeaux, France; 13 Laboratory of Hematology and Flow Cytometry, CHU Lyon-Sud Hospital, Hospices Civils de Lyon, France; 14 Division of Hematology/Oncology, Department of Medicine, Weill Cornell Medical College, New York, USA; 15 Department of Hematology, Medical University of Lodz, Poland; 16 Department of Hematology and Bone Marrow Transplantation, Wroclaw Medical University, Wroclaw, Poland; 17 Department of Hematology, Hemostasis, Oncology and Stem Cell Transplantation, Hannover Medical School, Hannover, Germany.; 18 Department of Internal Medicine III, Ulm University Hospital, Ulm, Germany.

## Abstract

Measurable residual disease (MRD) quantified by multiparameter flow cytometry (MFC) is a strong and independent prognostic factor in acute myeloid leukemia (AML). However, several technical factors may affect the final read-out of the assay. Experts from the MRD Working Party of the European LeukemiaNet evaluated which aspects are crucial for accurate MFC-MRD measurement. Here, we report on the agreement, obtained via a combination of a cross-sectional questionnaire, live discussions, and a Delphi poll. The recommendations consist of several key issues from bone marrow sampling to final laboratory reporting to ensure quality and reproducibility of results. Furthermore, the experiences were tested by comparing two 8-color MRD panels in multiple laboratories. The results presented here underscore the feasibility and the utility of a harmonized theoretical and practical MFC-MRD assessment and are a next step toward further harmonization.

## INTRODUCTION

The presence of measurable residual disease (MRD) in acute myeloid leukemia (AML) is an independent prognosticator of relapse and shorter survival.^1–5^ MRD can be measured at high sensitivity using molecular assays (RT-qPCR, next-generation sequencing [NGS]) or multiparameter flow cytometry (MFC).^4–7^ Advantages of MFC-MRD are its wide applicability (>90% of AML cases), short turnaround time (TAT), relatively high sensitivity (10^−3^ to 10^−5^), and the possibility to discriminate between living and dead cells.^8^ Nevertheless, this technique has recognized flaws: (1) sensitivity and specificity depend on the different monoclonal antibody (MoAb) panel, numbers of cells analyzed, and discriminatory level of the leukemia-associated immunophenotypes (LAIPs); (2) there is limited harmonization and standardization, since many laboratories use their own MFC-MRD assay; and (3) analysis and interpretation of data require relevant expertise. Taken together, these may limit comparability and clinical interpretation of MFC-MRD results. Therefore, an international group of experts on behalf of the European LeukemiaNet (ELN) proposed in 2018 consensus recommendations regarding the entire process from specimen collection to result reporting.^5^ Here, we provide more in-depth and updated technical guidance for flow cytometric AML-MRD from the ELN expert group following live ELN working group discussions, interlaboratory comparisons, a cross-sectional survey and a Delphi poll, all conducted between 2019 and 2021 (details in Supplementary Material, Supplemental Digital Content, http://links.lww.com/HS/A214 and Heuser et al^9^). These addressed how specific technical procedures can affect the quality of the flow data, aspects of assay analytical performance (eg, limit of blank [LOB], limit of detection [LOD], lower limit of quantification [LLOQ]) and included evaluation of 2 candidate 8-color MRD panels designed to facilitate standardization.

## SAMPLE PREPARATION

### Bone marrow sampling procedures

The quality of the bone marrow (BM) aspirate depends substantially on the skill and experience of the person who performs the procedure. MRD should be assessed from a small volume (<5 mL) of the first pull of a BM aspirate to prevent dilution from peripheral blood (PB) (hemodilution). In fact, MRD frequencies are about 1-log lower in PB compared with BM, causing an increased likelihood of false-negative results when the sample is diluted.^10,11^

Hemodilution has emerged as a critical issue to assess the reliability of a MRD test. There are several modalities to detect it, mainly consisting of formulas to detect PB contamination (Table 1).^12–16^ All formulas require additional measurements to estimate hemodilution or BM purity, such as matched PB or specific markers, which are not included in the standard MRD panels. Easier to implement is the examination of the mast cell population (CD117^hi^), as a decrease (≤0.002%) might indicate PB contamination^15^; however, the frequency of mast cells may be altered in myeloid neoplasias.^17^ Another option to estimate the possible contamination of PB is by determining the proportion of mature neutrophils, with a presence above 90% indicating hemodilution.^5^ An alternative approach is to change the denominator of the MRD assay from white blood cells (WBC) to the primitive/progenitor fraction (PM-MRD: based on CD34, CD117, or CD133).^5,18^ No consensus has been reached for a specific modality to be used in daily practice because of implementing difficulty due to TAT or costs in large centers with many samples to process on a daily basis. However, consensus was that all used strategies should be further explored by the different centers to assess the frequency and extent of hemodilution in the different clinical trials. When the potential relevance has been established, it can be implemented as a standard comment of sample quality for MRD reporting. For now, when hemodilution is documented (including hypocellular, nonregenerated BM), a second BM evaluation should be requested within 2 weeks to avoid unreliable MRD results.

**Table 1.** Overview of Formulas Proposed for Calculating Hemodilution

Formula for Detecting Hemodilution	Additional Requirements
**Bone marrow purity = [1-(erythrocytes BM/erythrocytes PB) × (leukocytes PB/leukocytes BM)] × 100%**	Matched PB^16^
**PB contamination index = −3.052 + 0.065 × (%CD10+ neutrophils of granulocytes) −0.609 × (%CD34+)−2.008 × (%plasma cells**)	CD10, CD34 positive cells and plasma cells^12^
**Normalized blast count = (80%/% dim CD16) × blast count**	CD16^13^
**Predicted bone marrow purity = [1 – (Lymphocytes FCM / Lymphocytes PB) × (Leukocytes PB / Leukocytes FCM)] × 100%**	Matched PB^14^
**Suggested blood contamination if mast cell population (CD117**^**+**^) ≤ **0.002%**	CD117 positive mast cells^15^

BM = bone marrow; FCM = flow cytometry; PB = peripheral blood.

**Figure 1.**
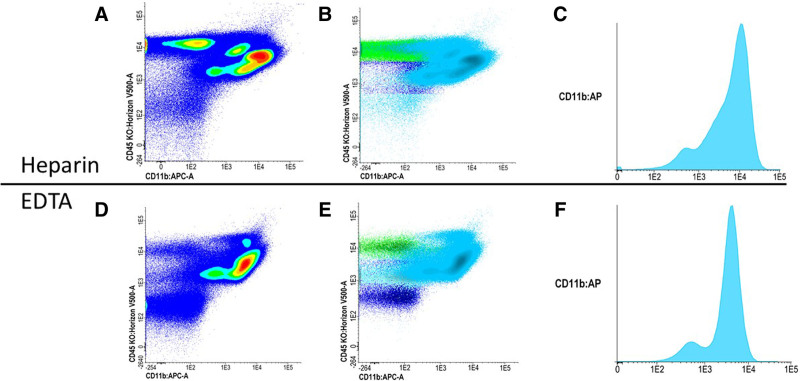
**Influence of anticoagulant.** (A, B, C) CD11b expression on a sample with heparin anticoagulant. (D, E, F) The same sample but now collected in an EDTA tube. One noteworthy influence of EDTA anticoagulants on a sample is the change of expression patterns of specific antigen, such as CD11b. CD11b expression diminishes with EDTA (D and E) compared to heparin coated tubes (A and B).

Another factor of variability is the anticoagulant used in tubes for MRD sample collection. Most ELN centers recommend EDTA tubes although heparin and sodium citrate are also utilized. All anticoagulants are liable to influence the sample. Although EDTA allows for a prolonged conservation of the samples over time,^19^ it may induce a change of expression patterns of antigens such as CD11b (Figure 1).^21^ Due to this, heparin has been recommended by the ELN myelodysplastic syndromes (MDS) workgroup.^22,23^ However, the other anticoagulants are acceptable as long as the laboratory validates its assay for stability.^24^

### Sample Transportation

**Figure 2.**
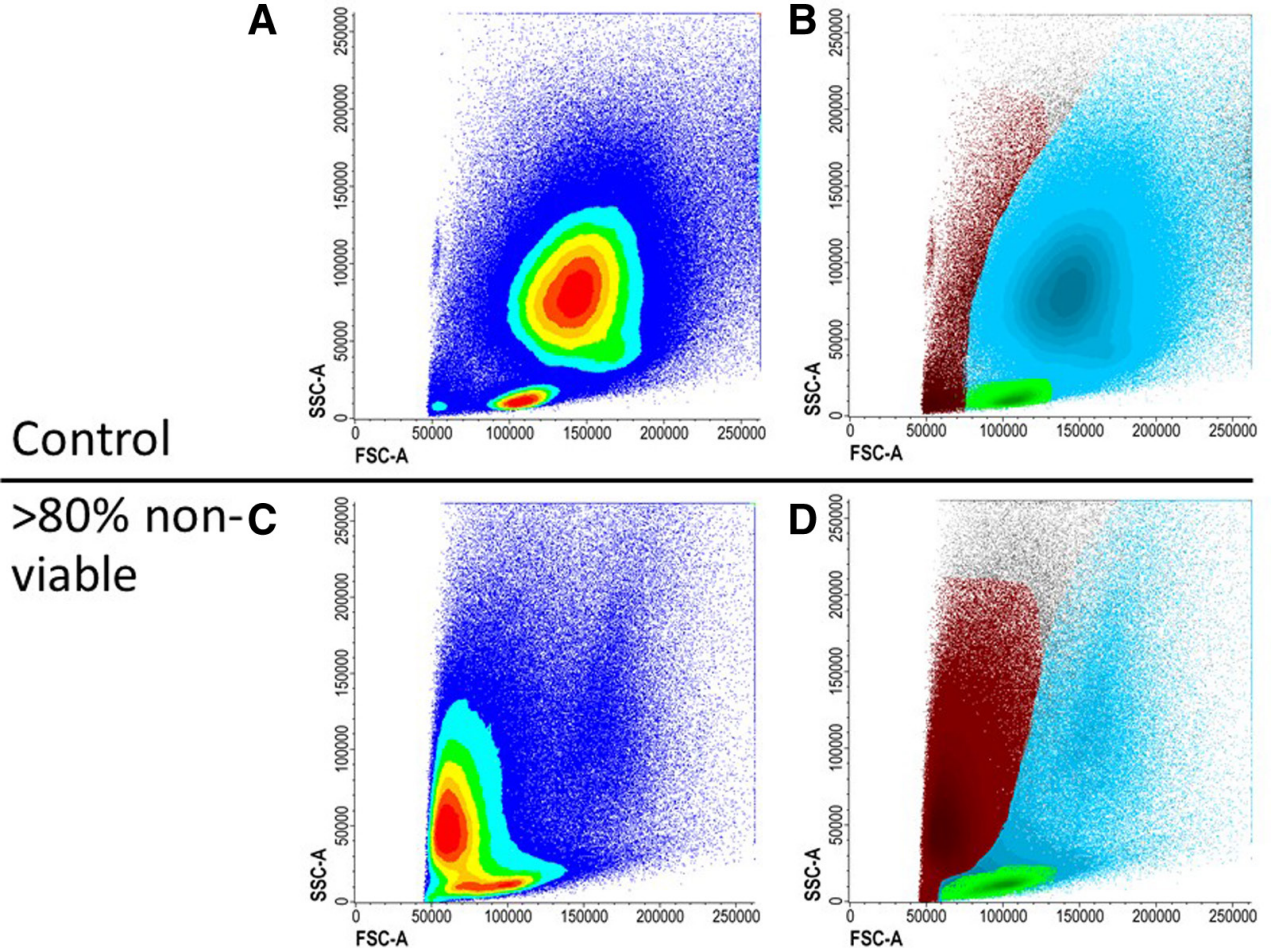
**Nonviable sample.** (A and B) A sample is preferably measured within 72 hours of collection. Prolonged time between collection and measuring will decrease the viability. (C and D) The sample is measured after approximately 240 hours after collection. More cells die causing FSC and SSC to diminish. FSC = forward scatter; SSC = side scatter.

From the ELN experience, most MRD testing is performed in centralized reference laboratories due to expertise, infrastructure, and cost. This generates issues related to sample transport. Since BM cells start to deteriorate once removed from the BM environment, the interval between BM aspiration and analysis should preferably be shorter than 72 hours.^25^ Samples are recommended to be stored at ambient condition to preserve cell viability.^26^ All samples are preferably tested for viability, stability, and overall quality, but this is particularly important if a sample is more than 72 hours old. Viability and overall quality of the sample is assessed, even if no viability dye is included, by the initial plotting of forward scatter (FSC) versus side scatter (SSC) that checks the light scatter properties of the sample at the beginning of gating strategies.^27^ When viability is inadequate based on the presence of high number of debris in FSC and SSC (Figure 2), it is advisable to request a second BM specimen. When BM sample quality appears suboptimal, MRD analysis can still be performed but should be accompanied by a comment on sample quality in the report.

### Sample processing

**Figure 3.**
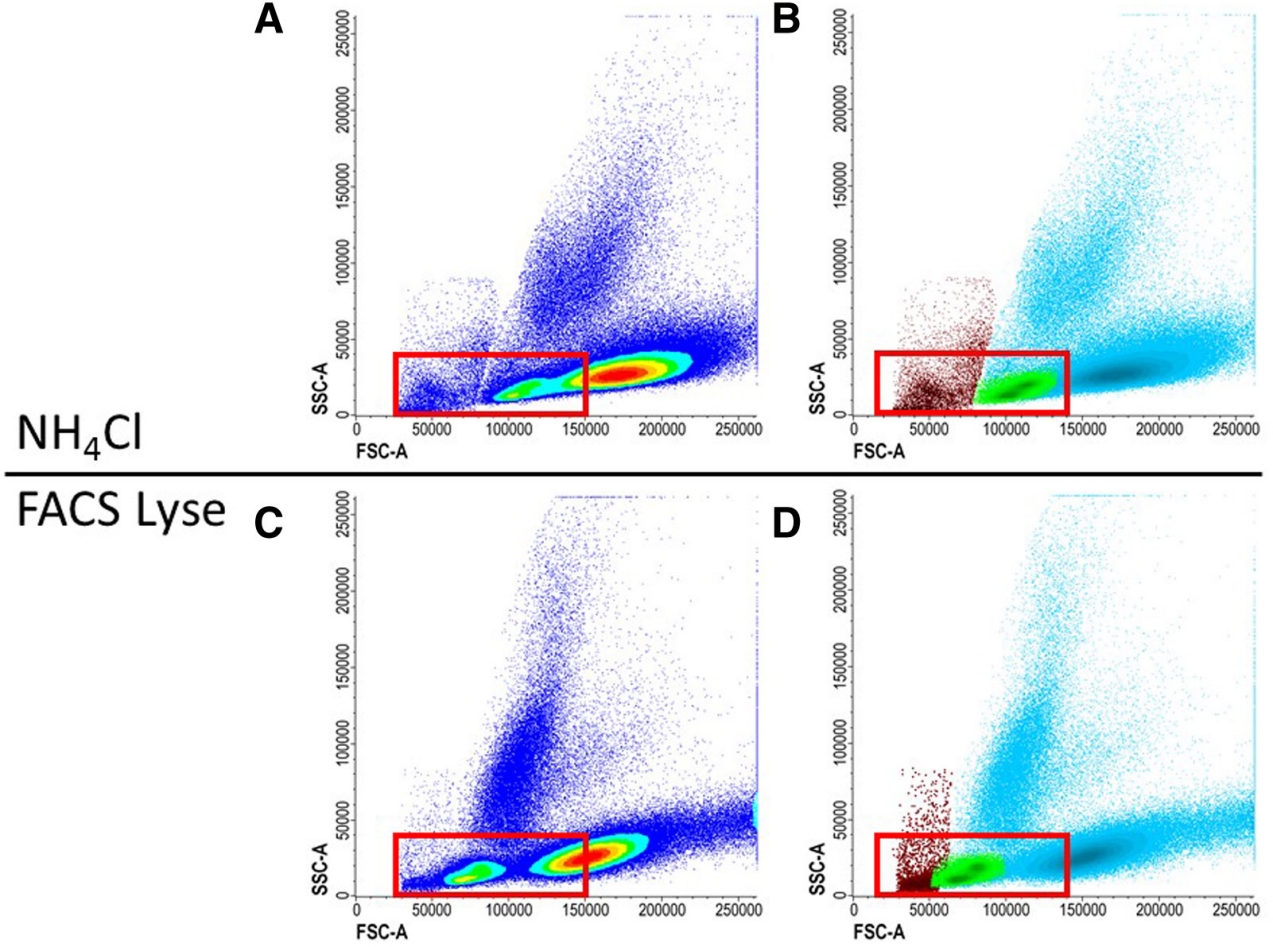
**Effect of lysis solution.** When choosing a lysis solution, FSC and SSC properties should be closely monitored to detect the introduction of artifacts. Lysis solutions can decrease the cell size and therefore decrease the FSC resulting in a poor distinction between red blood cells, lymphocytes, and WBC. (A and B) The distinction between WBC (blue), lymphocytes (green), and red/dead cells (red) are clearly seen with ammonium chloride (NH_4_Cl) as lysis buffer. (C and D) The same sample is lysed with FACS Lyse sample, but other settings remained the same, resulting in less difference between cell populations. FACS = fluorescence-activated cell sorting; FSC = forward scatter; SSC = side scatter; WBC = white blood cells.

There are 2 main approaches to prepare a BM sample for MRD-MFC acquisition. The first and most utilized procedure is stain-lyse-wash (SLW), where the washing step can be omitted.^28,29^ This method starts with staining using the appropriate amount of MoAbs, followed by lysing and optionally washing with phosphate-buffered saline (PBS). Human serum albumin or other protein solutions (0.1%) can be added to PBS to prevent cell clumping or cell aggregation to plastic, especially relevant for samples with poor quality such as those processed >72 hours after aspiration.^30^ The second approach is lyse-wash-stain-wash (LWSW) procedure. This includes bulk lysis of red blood cells followed by washing with PBS, resuspension of the pellet in a smaller volume allowing for increased cell concentration, staining of the cells with MoAb cocktail, and sometimes a second washing with PBS.^30^ The bulk lysis may also lead to a higher reproducibility, since the labeling conditions are reproducible, and the volume is constant for a given quantity of cells. The SLW procedure allows a clearer separation between positive and negative events, while using the bulk lysis followed by washing allows leukocytes to be concentrated and causes fewer artifacts.^29,31^ Different lysis solutions are used to eliminate red blood cells. Ammonium chloride (NH_4_Cl) preparations show minimal effects on WBC counts, however the washing steps required to remove the buffer yield unavoidable cell loss. Lysis buffers, may contain chemicals to fix remaining WBC after red blood cells lysis, for example, fluorescence-activated cell sorting Lyse (BD Biosciences, San Jose, CA), contains approximately 1.5% paraformaldehyde at diluted working solution concentration. These effects may also be achieved by adding a small amount (0.25%) of ultra-pure formaldehyde to NH_4_Cl buffer in an SLW procedure to allow fixation with preservation of light scatter properties allowing viability assessment and preservation of nucleated red cells.^27^ Several other stable commercial lysis reagents such as Versalyse (Beckman Coulter; Miami, FL) allow omitting washing without damaging leukocytes. The panel does not suggest a specific lysis protocol, provided that the selected lysis solution has the ability to maintain optimal FSC and SSC properties, and the mean fluorescent intensity (MFI) of all markers that should allow for the detection and enumeration of cell populations of interest (Figure 3). The advice of the ELN MFC-MRD working group is against additional sample fixation, as it causes changes in morphology due to cell shrinkage and loss of granulation, resulting in worse FSC and SSC.^25^ The same sample preparation should be applied to all MRD samples regardless of the time point.^29^

## FLOW CYTOMETRY

### Monoclonal antibody panels

Current MoAb panels used by ELN members consist of 8 to 10 fluorochromes to allow proper discrimination of the aberrant markers required to identify MRD. The ELN MFC expert group agreed on specific MoAb backbone markers to select cell populations of interest, to improve comparability among labs, and to allow, by specific software, merging of different tubes with various LAIP markers. The backbone should consist of 3–5 markers among CD45, CD34, CD117, CD13, CD33, or HLA-DR. This combination provides CD45 for WBC gating, primitive markers (CD34, CD117), and myeloid markers (CD13, CD33, or HLA-DR) to highlight the leukemia cell population. Additional markers will comprise the LAIP-specific antibodies (eg, CD56 and CD7), which are most informative for distinguishing leukemic cells from normal hematopoietic cells.^32^ This recommendation was already included in the previous version of the guidelines, but in this paper, it is also substantiated by a multicentric validation of a tube including these backbone markers. In cases with a monocytic/myelomonocytic component (5%–10% of AML cases, but more often seen in high-risk MPN/MDS), CD64, CD11b, and CD4 may be added, such as in the previously proposed special “monocytic tube,” consisting of CD64/CD11b/CD14/CD4/CD34/HLA-DR/CD33/CD45.^5,33^ This tube relies mostly on lack of CD14 expression on CD4^+^HLA-DR^+^CD64^+^ monocytic cells or lack of HLA-DR, CD4, and CD64 on CD14 positive cells. Other markers can be explored in this specific setting (eg, CD56, CD35, IREM2) as well.^34^

### Cytometer settings and set-up of the flow cytometry instruments

Standardized flow cytometer settings are crucial for reproducible measurement and should avoid interference between fluorochromes. To make comparable measurements between different instruments, standard flow cytometer settings for target MFIs are frequently used, such as the EuroFlow^29^ and the Harmonemia settings.^35^ Daily cytometer calibration checks are strongly advised to verify correct performance of the cytometer’s optical configuration.^36^ In addition, it is recommended to harmonize instrument settings using calibration beads specific to the instrument platform. Specialized reference beads are also used on the cytometer to calibrate and standardize performance to reduce intertest variability.^37^ Given the increasing number of fluorochromes available for standard instruments and accompanying increased complexity, automated compensation should be performed using either software supplied with the instrument or off-line software. Manual compensation is strongly discouraged by the panel because it is time-consuming and can cause incorrect/irreproducible MFC results.^25^

### Sample running and leukemic cell detection

**Figure 4.**
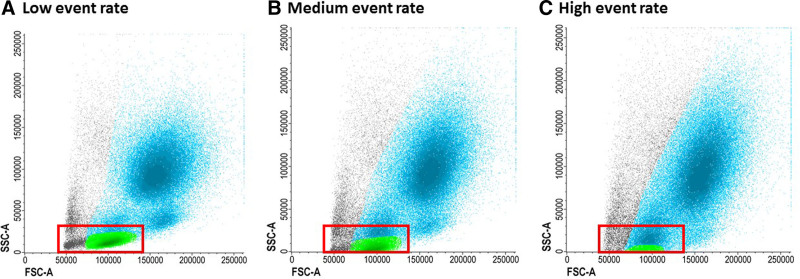
**Increased event rate.** The duration of measuring can be reduced by increasing the event rate. (A) The events acquired should clearly be separated from the y-axis (SSC-A). (B) However, increasing the event rate can affect the result by reducing the SSC, resulting in a distorted picture and hamper interpretation of expression patterns (B and C). SSC = side scatter.

**Figure 5.**
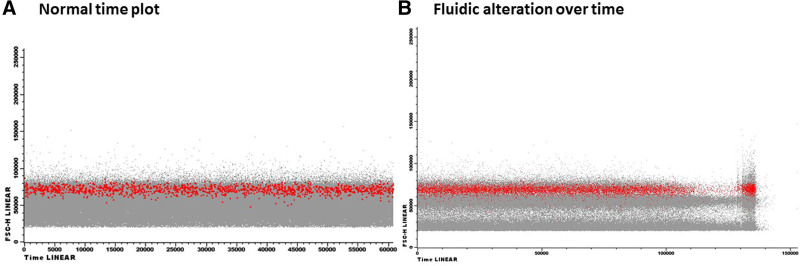
**Disturbances in time.** (A) Example of similar amount of events acquired over time. (B) Turbulences or disturbances may substantially alter the detection of rare events. Therefore, plotting time against parameters sensitive to fluidic alterations could allow to perform postacquisition corrections during analysis.

At diagnosis of AML, the BM contains frequent LAIP+ blasts and these high numbers allow accurate LAIP detection by acquiring at least 50,000 events in the blast gate. By contrast, MRD events in follow-up samples are expected to be scarce and the process should follow the rules of rare events acquisition. The sensitivity of the assay depends on the number of relevant events acquired. To allow accurate MRD assessment at the limit of detection (LOD) and limit of quantification (LOQ), acquisition of a minimum denominator of 500,000 to 1,000,000 CD45-expressing events using the combined LAIP/DfN (different from normal) approach is advised and negativity should be confirmed with all tubes. Increasing the number of analyzed cells may further improve sensitivity and allow monitoring of minority clones identified by diagnostic LAIPs. Acquisition at a high pressure or flow rate can lead to several technical issues; for example, changes in scatter patterns may disturb the gating procedure (Figure 4). Furthermore, fluidic instability, for example, shear turbulence or disturbances, may substantially alter the detection of rare events; therefore, plotting the TIME parameter against any other sensitive to fluidic alterations allows postacquisition correction during analysis (Figure 5). Today, events not fulfilling appropriate characteristics of singularity, compensation, and fluidic may be excluded from the final analysis by dedicated softwares (eg, FlowAI).^38^

### Selection of control samples

To define whether antigen expression is aberrant, it is essential to exploit every possible effort in evaluating the performance of each aberrant phenotypes under different conditions. This reference is generated by running the selected MRD panel on sufficient numbers of normal (“control”) BM samples, collected during surgical procedures, bone marrow harvesting from healthy donor, or in patients with solid or hematological malignancies but without BM infiltration. The number of control samples should be at least 10 and the procedure should be repeated every time the assay methodology is modified. A larger number and variety of samples will minimize the risk of “control” samples containing aberrant cells that could be mistaken for AML. It is agreed that obvious outliers in control BM, for example, >2 SD, should be excluded. Also, during “stressed” normal differentiation, antigen expression can change without implying the presence of disease. Furthermore, in older patients clonal hematopoiesis of indeterminate potential can occur, although its influence on differentiation antigens pattern is not fully elucidated.^39,40^ However, there is some evidence that preleukemic clonal hematopoiesis may persist in AML patients who are in complete remission without any genetic evidence of MRD, and that the aberrant blast phenotypes may represent preleukemic clonal hematopoiesis leading to their misidentification as MRD.^41,42^ Age-matched controls are therefore preferred to avoid possible age-related differences in marker expression to be identified as LAIPs. Furthermore, it is essential to know how the surface marker expression may be modified in the recovery phase after chemotherapy or transplantation. This observation implies that marker combinations defining a LAIP may have different frequencies in normal background marrow cells and, consequently, different specificity for MRD detection.^43^ Several sources can be used to investigate BM regeneration such as samples after completion of consolidation therapy with no subsequent emerging leukemia; or post stem cell transplantation LAIP negative patients with no subsequent relapse or patients treated with myelosuppressive chemotherapy for other malignancies not involving BM.

## DATA ANALYSIS OF FLOW CYTOMETRY DATA TO ASSESS AND REPORT MRD

### Gating strategy

The identification of leukemic cells starts by visualizing the different cell populations, irrespective of the software used. To ensure the best quality of relevant event acquisition, a parameter sensitive to fluidic alterations [eg, FSC-Height or FSC-Area] should be displayed versus TIME in each tube. After eliminating debris and checking for viability on an FSC/SSC scatter plot, as mentioned earlier, it is recommended to perform doublet discrimination (eg, FSC-A versus FSC-H) to exclude cell aggregates. Then WBC are defined as the CD45 expressing population in a CD45/SSC plot. The primitive/progenitor cells can be found in the CD34+, CD117+, or CD133+ fractions. The immature blasts may be found in the CD45dim/SSClow population or in the CD45 high/CD34 negative fraction if cells are more mature. Within those fractions, aberrancies can be defined when myeloid markers (CD13 and CD33) are combined with lineage markers not seen in normal bone marrow (eg, CD7 and CD56). Combinations between the primitive marker positive cells and maturation markers can also be found (eg, CD11b). In addition, some under- and over-expression of markers can be observed (eg, CD33 and HLA-DR). Since the MRD gating strategy requires hierarchical or sequential gating to identify the aberrant immunophenotype, a final confirmation of complete inclusion of the leukemic population in each gate employed is suggested. This can be usefully visualized using a density plot display. As a final step, backgating of the LAIP cells on the CD45, CD34, and SSC/FSC plot can be used to ensure identification of appropriate population. Backgating of CD45 and FSC plot are especially important for monocytic/myelomonocytic aberrancies, which are harder to gate compared to immature cells due to the high overlap with regenerating bone marrow. The gating strategy is visualized in Figure S1, Supplemental Digital Content, http://links.lww.com/HS/A214.

### Data analysis and interpretation

Different software can be used for MRD-MFC analysis of the digital data files (.fcs) with similar performance. Every software has its own advantages and disadvantages. There is no agreement to the minimal percentage of LAIP that should be present at diagnosis to select a given marker for MRD monitoring during therapy. Since antigen expression can shift during therapy, it is recommended to identify more than 1 LAIP at diagnosis and to recognize the presence of new LAIP following therapy to reduce false-negative results when using the LAIP approach. In principle, the LAIP method measures the most prominent LAIP population at diagnosis, which is then followed during therapy.^30^ This approach is optimized by the analysis of a baseline sample at diagnosis, which is not always available in daily routine settings but should be strongly recommended. In addition, emergent clones can result from clonal evolution or from the persistence of chemoresistant subclones during follow-up and might be missed with the LAIP approach.^44–46^ Therefore, some researchers use the DfN approach to identify aberrant populations following therapy.^27^ The DfN approach can be applied in the absence of a diagnostic sample. Since it does not rely on stability of the diagnostic LAIP during treatment course it can identify leukemic populations even when a subclone emerges outside the LAIP selected at diagnosis. However, this approach requires detailed knowledge of normal and regenerating BM profiles to distinguish the AML clones from preleukemic immunophenotypes.^41^ The different techniques also influence the specificity and sensitivity of the assay, with likely higher specificity of the LAIP method compared to higher sensitivity with the DfN approach.^43^ The ELN experts would therefore recommend a combination of the 2 analysis methods,^5^ recognizing that caution should be used for certain markers that may be transiently expressed in regenerating BM (eg, CD25, CD22, and CD15, from published and shared experience).^43^ Regardless of the approach used, the panel suggest a particular attention be devoted to emerging clones also in regions not originally included in the blast gate. An example of an emerging clone in the DfN approach can be found in Figure S2, Supplemental Digital Content, http://links.lww.com/HS/A214.

For clinical decision-making based on the presence of MRD, the threshold defining positivity is of crucial importance and may depend on the time point of sample collection during and after therapy, the treatment schedule and the AML subtype. In general, the threshold of 0.1% after 2 cycles of chemotherapy is used as a prognostic factor in AML for outcome and for clinical decisions regarding intensity of the consolidation treatment.^47^ Although more research is required to validate the cutoff for a particular clinical situation, it is essential to be aware of the performance characteristics of the assay in the lower ranges of detection due to the interference of background events. For accurate knowledge of assay performance, 3 characteristics need to be considered: (1) LOB is the maximum number of LAIP cells measured in samples lacking leukemia (such as normal or regenerating BM or samples not stained with the antibody of interest) [LOB = mean_blank_ + 1.645(SD_blank_)]; (2) limit of detection (LOD) is the minimal number of LAIP cells that can accurately be distinguished above background [LOD = LOB + 1.645(SD_low positive_)]; (3) LLOQ is the lowest LAIP% that can be reliably quantified relative to a defined acceptance criterion and is equal to or higher than LOD.^48^ LOD is ideally established by measuring 10 samples having a very low positive LAIP in triplicate. A coefficient of variation (CV) of <30% is proposed to confirm acceptable LLOQ.^49^ The LOD of DfN gates is estimated in the same manner as for the LAIP approach.

For multiple myeloma and CLL MFC-MRD, a precise number of events to define the LOD and LLOQ are suggested based on CV calculated from Poisson statistics for rare events.^50,51^ It should be emphasized that these represent theoretical estimates of the reproducibility of enumeration for small numbers of events and must be compared with a desired criterion for reproducibility to determine a theoretical LOD or LLOQ, for example, a desired CV of <10% would require at least 100 events in the population. In addition, they do not take into account the impact of noise or other assay performance characteristics so are a best-case scenario that must be confirmed through experiment for each assay. Applied to AML-MRD assessment, a cluster of 20 events carrying an aberrant phenotype can be sufficient for the recognition of MRD in a well-controlled assay and can represent the LOD, that is, whether an abnormal population is present or not. Similarly, a cluster greater than 50 events can be regarded as the threshold for a standardized and reproducible enumeration of rare populations and can represent LLOQ if a CV of 14% is judged acceptable.^52,53^ Thus, the acquisition of 500,000 to 1,000,000 events will allow a theoretical LOD of 0.004% and 0.002% and a LLOQ of 0.01% and 0.005%, respectively. Note that the values obtained by this approach will often differ from those obtained using data as defined in the prior paragraph. These and other approaches are currently being evaluated for AML. The FDA advises to technically validate the LLOQ of the assay 1-log below the chosen threshold for clinical decision-making.^54^ This restricts lowering the threshold for MRD positivity and negativity that can be used in clinical studies, although lower MRD thresholds have been prospectively validated for prognostic impact as has MRD positivity defined as any detectable MRD.^6,55–58^ The panel did not modify the suggestions given in the first release of the guidelines, so, after 2 cycles of intensive chemotherapy, the threshold of 0.1% on a denominator of 500,000–1,000,000 CD45-expressing relevant events is still the standard to be pursued. This aside, LOD and LOQ of each determination should be specified and the clinical value of MRD above or below these limits should be actively researched.

### Reporting

In most multicenter clinical trials, the final MRD assay result is reported as “MRD-positive” or “MRD-negative” to the clinicians which makes the MRD results easy interpretable. However, this does not imply that all MRD detectable by the assay but below the “MRD positive” threshold (such as 0.1%) holds no prognostic significance. The “technical MRD” below the 0.1% threshold with appearance of residual or emerging leukemic populations may also be described in the report to alert the clinician in case monitoring closely with short follow-up is advisable. For clinical decision-making, the MRD assay should be analytically validated based on the guidelines for rare events in MFC.^59^ The accuracy and level of confidence for an MRD result is in part dependent on the number of relevant events acquired (CD45-expressing or mononuclear cells), as described earlier, so report results should be qualified if insufficient cells are evaluated to meet the assay’s validated LOD. In addition, report results should be qualified if sample quality, cell viability, or hemodilution impair the performance characteristics of the assay and may result in a false-negative result. When a sample does not meet the required quality criteria, a repeat BM sample should be requested in 2–4 weeks when clinically indicated. The diagnostic conclusion of MRD testing and the informative value of the final amount of residual leukemic cells should be mutually agreed upon between experts from the laboratory and clinicians. When molecular MRD is also available, the results should be combined to further increase the probability of predicting relapse.^55,60^

## INTERLABORATORY VALIDATION

**Figure 6.**
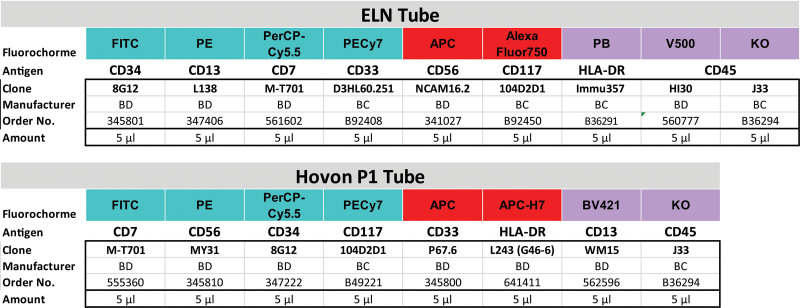
**Details of ELN tube and HOVON P1 tube composition.** (A) The ELN tube is designed based on the most common LAIPs and expertise of the ELN consortium as reported in Schuurhuis et al.^5^ There are 2 clone options for the CD45 antigen. (B) The HOVON P1 tube composition consists of the same fluorochromes but has different clones and antigens in different channels. ELN = The European LeukemiaNet; LAIPs = leukemia-associated immunophenotypes.

**Figure 7.**
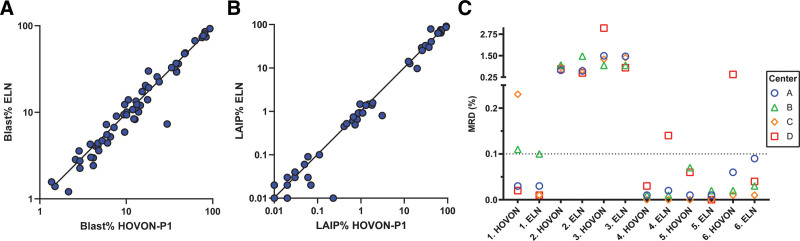
**HOVON-P1 and ELN tube comparison.** (A) Comparison of log-transformed blast percentage of 62 samples (both diagnose and follow-up) between HOVON-P1 tube (the first tube used from the Heamato Oncology Foundation for Adults in the Netherlands) (x-axis) and the ELN tube (y-axis) with a correlation coefficient of r = 0.99 (*P* < 0.001). (B) LAIP percentage compared on the same 62 samples as panel A (r = 0.98, *P* < 0.001). (C) Measurable residual disease (MRD) percentage (y-axis; split into 2 segments on MRD 0.25%) of all 12 follow-up samples per center. A total of 6 follow-up samples are measured with 2 different tubes (HOVON P1 and ELN tube). The centers and patient data are anonymized. The associated samples are next to each other on the x-axis with corresponding numbers. Samples used are at different time points during therapy and with different cytogenetic alterations. Three (HOVON sample 1 and 6; ELN sample 4) of the 12 samples did not have an anonymous MRD result between the 4 centers if the 0.1% cutoff would be used, although not all samples were collected after induction therapy. These differences could be explained when LAIP gates were individually examined. Other 9 samples had identical MRD result (4 MRD+ and 5 MRD–). ELN = The European LeukemiaNet; LAIPs = leukemia-associated immunophenotypes; MRD = measurable residual disease.

The working group proposes that a minimum set of CD markers/fluorochromes should be implemented as a prerequisite for harmonized MRD detection including 5 of the suggested (see earlier) backbone markers (CD45, CD34, CD117, CD13, and CD33), 2 aberrant lineage markers (CD7, CD56), and HLA-DR. To compare data from different laboratories using these markers, a consensus ELN tube based on a large German study was validated. In addition, the clinically validated HOVON P1 tube, which is validated in several HOVON/SAKK/AML-SG protocols, was used as comparison. With both tubes, experts could identify a useful LAIP in a range of 70%–90% of AML cases in different studies compromised of several hundred patients consisting of all WHO-AML subtypes except acute promyelocytic leukemia.^61^ Both tubes consist of the same antigens but with different fluorochromes measured in different detectors (Figure 6). There are 2 different CD45 clones available for the ELN tube depending on the instrument used: HI30 MoAb (order number 560777) for BD Biosciences instruments (San Jose, CA) and J33 antibody (order number B36294) for Beckman Coulter instruments (Brea, CA). Both tubes were measured in a subset of 62 diagnosis and follow-up samples by 4 different laboratories, which showed that blast percentage (R_pearson_ = 0.99, *P* < 0.001) and MRD-percentage (R_pearson_ = 0.98, *P* < 0.001) were significantly correlated (Figure 7A, B). Moreover, we selected 6 samples, which were measured in 1 laboratory using both HOVON P1 and ELN tubes and compared the results solely based on the gating between 4 centers with 3 different methods of gating including a DfN approach. Parameters collected were as follows: WBC, blast-, and MRD-percentage. Although a good concordance in MRD% was found between the tubes in each laboratory, there were some outlier results (in 5/24 analyses), occurring equally in both tubes (Figure 7C). After discussion by reviewing the results together, it was agreed that the gating of CD45 and CD34 is crucial and the CD34 positivity was a little more distinct in the HOVON tube. As shown in the figure, some outliers were present in this comparison, but these were revoked after discussion by adjusting the gates and in 1 case by selecting another CD marker combination as LAIP. The presented results reflect the need for strict gating strategies and definition of useful LAIPs to have the best comparison between different laboratories. The most often found LAIPs using these tubes are a combination of CD34 or CD117 as primitive marker with: CD7+, CD56+, CD33–, and CD13–/CD33+ (an overview of most used LAIPs from the consensus tube can be found in Table S1, Supplemental Digital Content, http://links.lww.com/HS/A214). A marker combination that was frequently seen in regenerating bone marrow and therefore not recommended is CD34-CD117+HLA-DR+.

## DISCUSSION

The number of centers using MFC as a tool to measure MRD is progressively increasing worldwide, and there is a concomitant demand for harmonization and qualification. This manuscript provides a consensus document for the optimal MFC-MRD assessments based on the combined multicenter expertise of the ELN-group members. MRD assessment using MFC should be performed in a specialized laboratory due to the high complexity of the analysis. Strict quality criteria should be put in place to ensure comparability of MRD results among laboratories and different clinical studies. Laboratories might have some room to adhere to their own local standard operating procedures, but baseline criteria are crucial. First, the flow cytometer settings should allow quality control and adequate scatter properties. Second, the chosen panel is recommended to contain backbone markers (recommended: CD45, CD34, CD117, CD13, CD33, and HLA-DR, see earlier) in each tube to clearly identify the cell populations of interest. The third requirement is a strict gating strategy that has been validated to measure LAIP cells not found in normal and regenerating BM. Finally, to use the MRD measurements for clinical decision-making, the assay needs to be fully validated (LOB, LOD, LLOQ, and other features such as precision and stability).

Lack of harmonization in technical approaches to MRD measurements may render MRD data comparison between studies challenging, as seen in an some meta-analyses.^62^ It may also reduce its prognostic value.^63^ Yet, recently a meta-analysis of 81 studies with different MRD methods showed a clear prognostic value for MRD in uni- and multivariate analyses.^4^ The implementation of a universal tube is a huge step to reduce interlaboratory variation. Both the ELN- and the HOVON P1 tubes allowed good distinction of different cell populations and produced comparable intra- and interlaboratory results. Large multicenter clinical trials have been performed using these tubes and when complemented with additional data, will allow further insights in optimization of MRD for clinical use. Although not accredited for AML-MRD and still in a pilot phase, the initiative of United Kingdom National External Quality Assessment Site to standardize MRD measured by flow cytometry and add an external quality assessment is much appreciated. The round robin test performed by the initiative is an example of how a 4-eye principle and harmonizing results can lead to improved MRD measurements.

Interpretation of these findings are included in an update of the AML-MRD consensus manuscript in which the recommendations were substantiated using a Delphi poll (See also Heuser et al).^9,64^ Interestingly, our expert panel on MFC-MRD had a mean overall consensus of 88% (range 76%–94%) in the last round of the Delphi poll. The main message of our intensive collaboration is, that we may not need to have 1 method that fits all but defined essential quality targets to achieve accurate and reliable MRD results.

### Future perspectives

There are several new factors that might improve MRD assessment in the near future. Currently, 25%–30% of MRD-negative patients still develop a relapse and some ways to reduce these false-negative results are being investigated. We might see a larger contribution of MRD based on leukemia stem cell (LSC) frequency because it has shown to be of prospective additive value to MRD in AML risk classifications.^65,66^ The largest drawback to LSC is the effort to identify the very low frequency of the stem cells, which may not be present in all subtypes of AML with similar immunophenotypes. Moreover, LSC population testing also has to undergo the same standardization/harmonization steps as MRD testing by MFC (eg, instrument settings, reagents and panel). One recent major step toward standardization is the fabrication of a single LSC tube,^67^ that has been validated in a multicenter setting.^68^ Also implementation of prepared dry tubes kits could limit technical variation, but research is still ongoing.

MRD assessment is mostly used for clinical decision-making after cycle 2 to select the appropriate consolidation and pretransplant therapy. There is less MRD assay data available to guide decision-making at other time points. It has previously been observed that the optimal time point for MRD measurement could be influenced by specific molecular aberrations.^69^ Across different studies, different cutoffs are used to define MRD positivity (0%–0.1%).^52^ The optimal cutoff is still under debate, in part because of the relatively high LOD and LLOQ of current assays. As the cutoff for clinical decision-making is lowered, LOD and LLOQ need to be lowered as well and more sensitive and consistent approaches must be developed. Background events will also play a larger role at lower MRD cutoffs, increasing the chance of a false-positive result. Therefore, although this is a time-consuming effort, it is strongly advised to determine the LOB/LOD/LLOQ and background expression mainly for the most used LAIPs to increase the accuracy of the assay.^43,45^ Likewise, as a complement to BM, the use of PB for monitoring of MRD could improve the accuracy of MFC-MRD because it suffers less from background.^10,70^ Single center studies showed that MRD as measured using PB is highly specific and may therefore have a prominent role in clinical management and MRD monitoring after treatment.^10,11^ Easier sampling could lower patient discomfort and allow MRD to be more frequently assessed. However, MRD monitoring can still be challenging in specific cases, such as AML with monocytic differentiation, where populations easily overlap, generating false-positive results. An extensive evaluation with cross-correlation is therefore warranted.

Recent developments in the field of precision medicine and targeted therapy for AML have led to an interest in finding specific molecular aberrations. New techniques, such as NGS may add further value in predicting the recurrence of leukemia.^60,71^ These techniques come with their own disadvantages and are to date less routinely applicable than flow cytometry but represent additional tools to increase the prognostic sensitivity and specificity of MRD detection.^72^

Currently, most laboratories still perform the gating strategy manually, which is claimed to be time-consuming, subjective, and expert dependent. Hence, an automated strategy with use of computational data to analyze the MFC-MRD diagnostics such as FlowSOM is promising and has gained a lot of interest.^20,73–75^ Unsupervised analysis has also been applied in the pretransplant setting.^76^ The future application of unsupervised/ machine learning approaches to data analysis may reduce interobserver variability and therefore contribute to the harmonization of MRD results.

## CONCLUDING REMARKS

In conclusion, MRD measured by MFC has become a relevant tool in an increasing number of hematology centers. Implementing techniques comes with challenges due to complexity, in particular for AML, in which clonal heterogeneity prohibits a “one size fits all” approach. In the future, consensus approaches, such as attempted in this paper, may contribute to reducing subjectivity and determine a common analytical backbone for MFC-MRD. This will facilitate interlaboratory comparisons of MFC-MRD results for daily practice and clinical trials as well as enabling future meta-analyses incorporating MFC-MRD big data.

### Take home messages

- MRD should be assessed from a small volume (<5 mL) of the first pull of a bone marrow aspirate to prevent hemodilution;
- Bone marrow samples should be stored at ambient condition and analyzed within 72 hours after collection and processed using SLW or LWSW procedures;
- At follow-up, collect a minimum of 500,000 CD45-expressing events and 100 viable cells in the blast compartment assessed for aberrancy(s) for determining MRD negativity;
- For clinical decision-making, MRD assessment should be performed with a qualified assay, including adequate LOB, LOD, and LLOQ with a harmonized use of the integrated LAIP and DfN strategy;
- Using these technical requirements with own in-house protocols, substantial concordance between different laboratories can be achieved as demonstrated with the consensus tube (composed of CD34, CD13, CD7, CD33, CD56, CD117, HLA-DR, and CD45).

## ACKNOWLEDGMENTS

We would like to thank the Dutch AML-MRD working party for their fruitful discussions relevant for these technical guidelines.

## DISCLOSURES

SF received speaker bureau for Pfizer, Jazz Pharmaceuticals. VB received research funding from Celgene; consultancy from Pfizer; consultancy and research funding from Gilead; research funding from Novartis; and consultancy from Amgen. AV received advisory role for Novartis, Pfizer, Jazz Pharmaceuticals, Amgen, Abbvie, Gilead, Astellas, Incyte, Janssen & Cylag, research funding to the Department of Biomedicine and Prevention, University Tor Vergata from Sandoz and Jazz Pharmaceuticals. LM received speaker bureau for Pfizer; advisory board for Abbvie, Janssen, Novartis and BMS/Celgene. RBW received advisory role for Amphivena, Astellas, Bristol Myers Squibb, Genentech, GlaxoSmithKline, Janssen, Kite, Kronos, and MacroGenics; ownership interests in Amphivena; research funding to institution from Amgen, Aptevo, Celgene, ImmunoGen, Janssen, Jazz, MacroGenics, Pfizer, and Selvita. AW received advisory for Abbvie, Astellas, BMS, Pfizer, Janssen, Novartis; honoria from Abbvie, Astellas, BMS, Novartis, and Janssen; research funding to institution—Jazz. MS received research funding from Seattle Genetics, Morphosys; consultancy and honoraria from Celgene; consultancy and research funding from Novartis; consultancy from Janssen; consultancy and honoraria from Pfizer; consultancy, honoraria, and research funding from Gilead Sciences; consultancy and research funding from Roche AG; consultancy, honoraria, and research funding from AMGEN. Other authors have no conflicts of interest to disclose.

## Supplementary Material


