# Supplementary material for: Technical Aspects of Flow Cytometry-based Measurable Residual Disease Quantification in Acute Myeloid Leukemia: Experience of the European LeukemiaNet MRD Working Party
Source: Hemasphere. 2021 Dec 22;6(1):e676. doi: 10.1097/HS9.0000000000000676 (PMC8701786; doi:10.1097/HS9.0000000000000676)
Supplement: Supplementary file 1 [file hs9-6-e676-s001.docx]

**Tettero et al. Supplemental material**

**Questions of questionnaire**

1. At which time point during AML disease do you assess MRD?
2. How do you handle the BM aspiration syringes?
3. Which anticoagulant do you use?
4. Which analysis time delay do you accept?
5. How is the quality of BM aspiration and potential hemodilution assessed?
6. What procedure is used If BM quality appears suboptimal?
7. What is your sequence of sample preparation for flow cytometry?
8. Which cytometer are you using?
9. Which number of events are acquired at diagnose and follow-up?
10. How many tubes (one tube contains e.g. 5 different antibodies) are measured for one follow-up sample?
11. How many AML MRD analyses are performed per year?
12. According to lab standards, certification, accreditation or QM, how often is a calibration of the cytometer performed? Which reagents are used?
13. Within your consortium, are instrument settings harmonized by using calibration beads?
14. Do you think standardization/harmonization of MRD assessment procedures is necessary?
15. Does the lab participate in AML MRD inter-laboratory tests?
16. Which Software do you use?
17. MRD AML assessment is done by which approach?
18. Which cut-off MRD level do you use?

**Description of the methodology used to reach consensus**

After several live discussions, a questionnaire of eighteen questions was send to all participating centers. Partly based on these results, twelve different consensus statements were drafted. These consisted of difference aspects of Multiparametric Flow Cytometry (MFC) MRD testing. A two-stage Delphi poll with a predetermined minimum of 70% level of agreement (LoA) per recommendation was conducted to optimize consensus. All members of the ELN AML-MRD working party could participate. Participants could rank all recommendations from ‘Completely disagree (1)’ to ‘Completely agree (5)’ and give specific comments per recommendation. Only ‘Agree (4) and ‘Completely agree (5)’ would count as approving the recommendation. After the first round, recommendations would be optimized or changed based on the input from participants or if the level of agreement was below the 70% LoA by two senior co-authors. Both the original and the (potential) changed recommendation were send again to all participants for rating. The recommendation with the highest consensus and which did not vary more than 10% between round 1 and 2 (stability of consensus), has been incorporated into this manuscript. An overview of all consensus statements with LoA can be found in the main ELN-guidelines of Heuser et. al. (manuscript in press).

**Figure S1.**


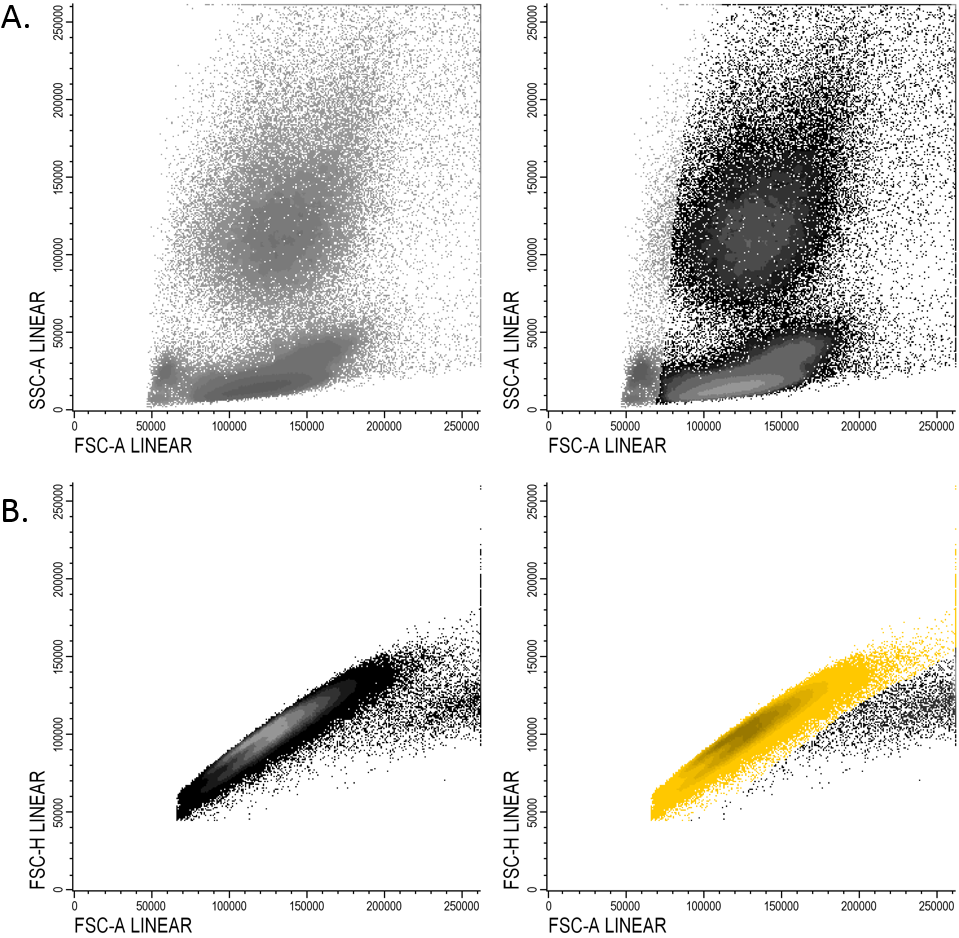


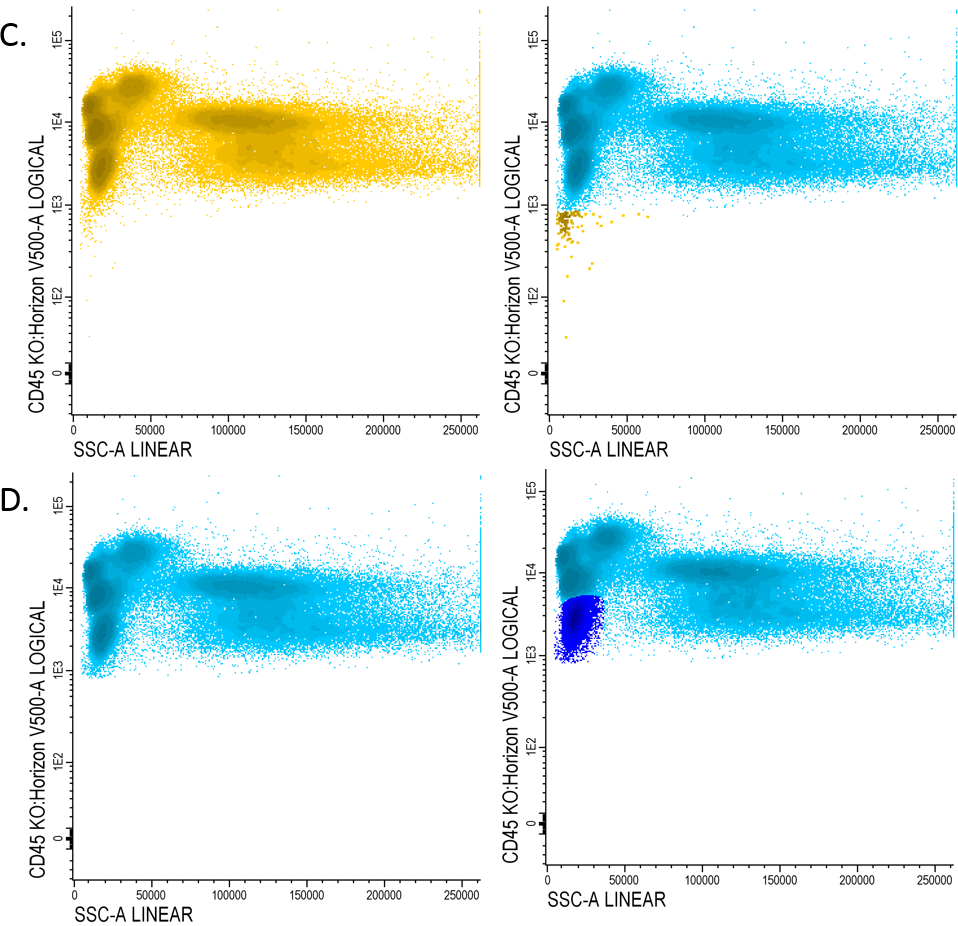


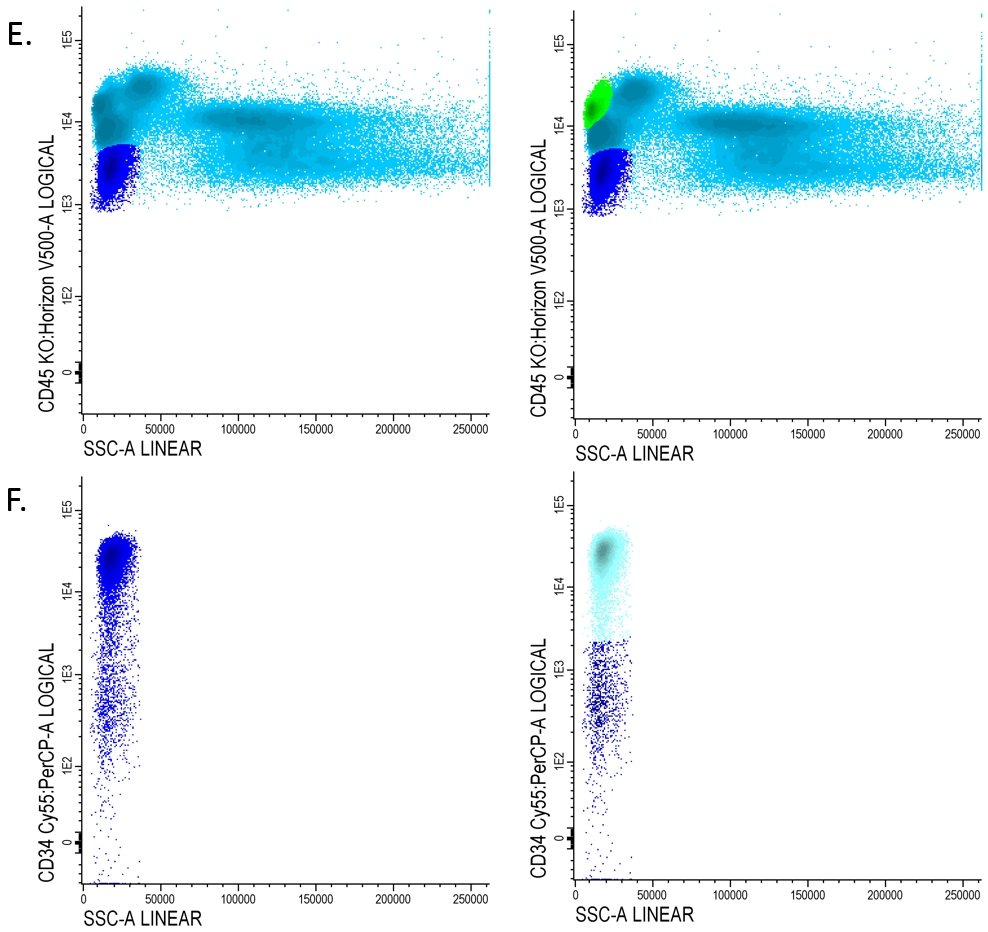


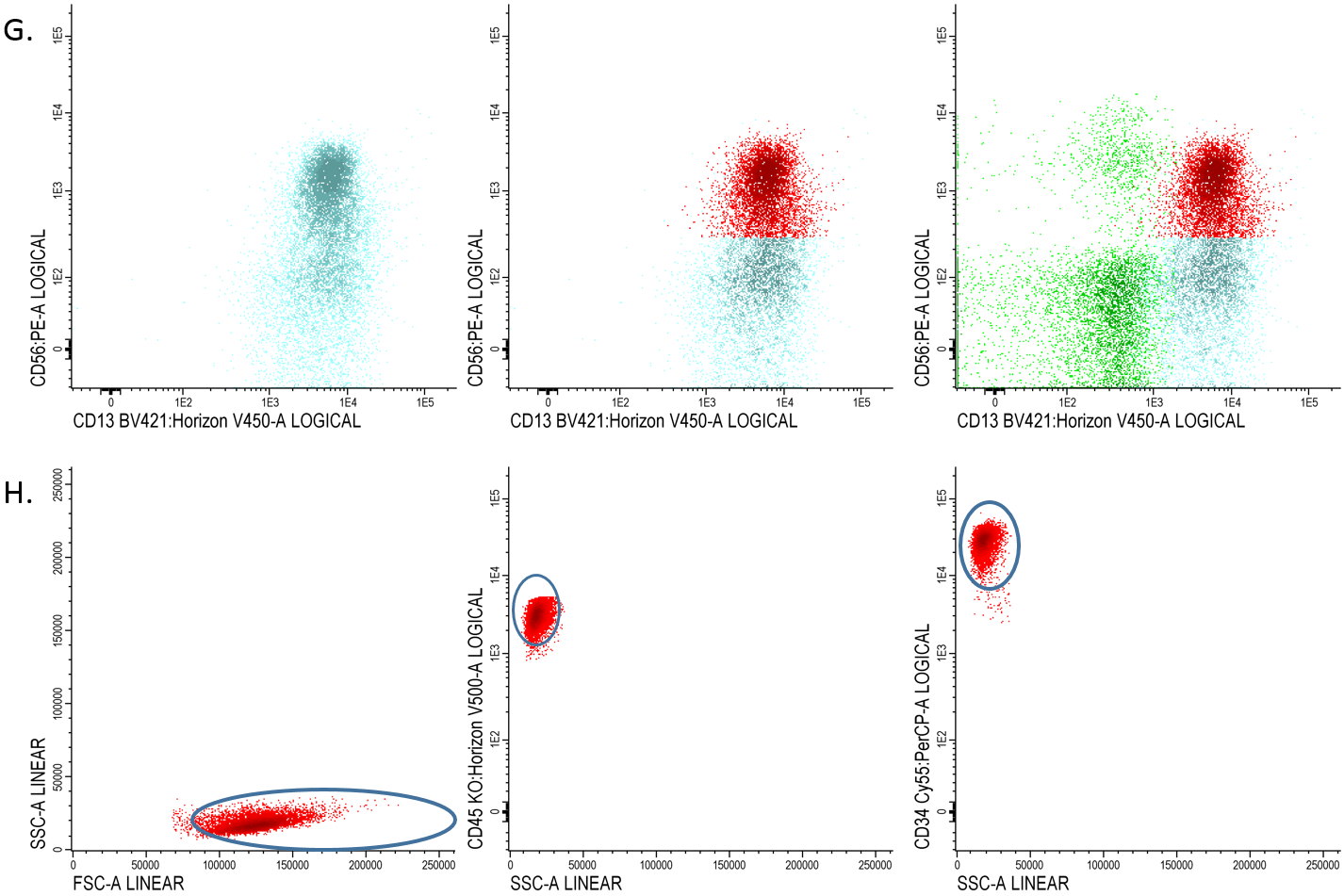


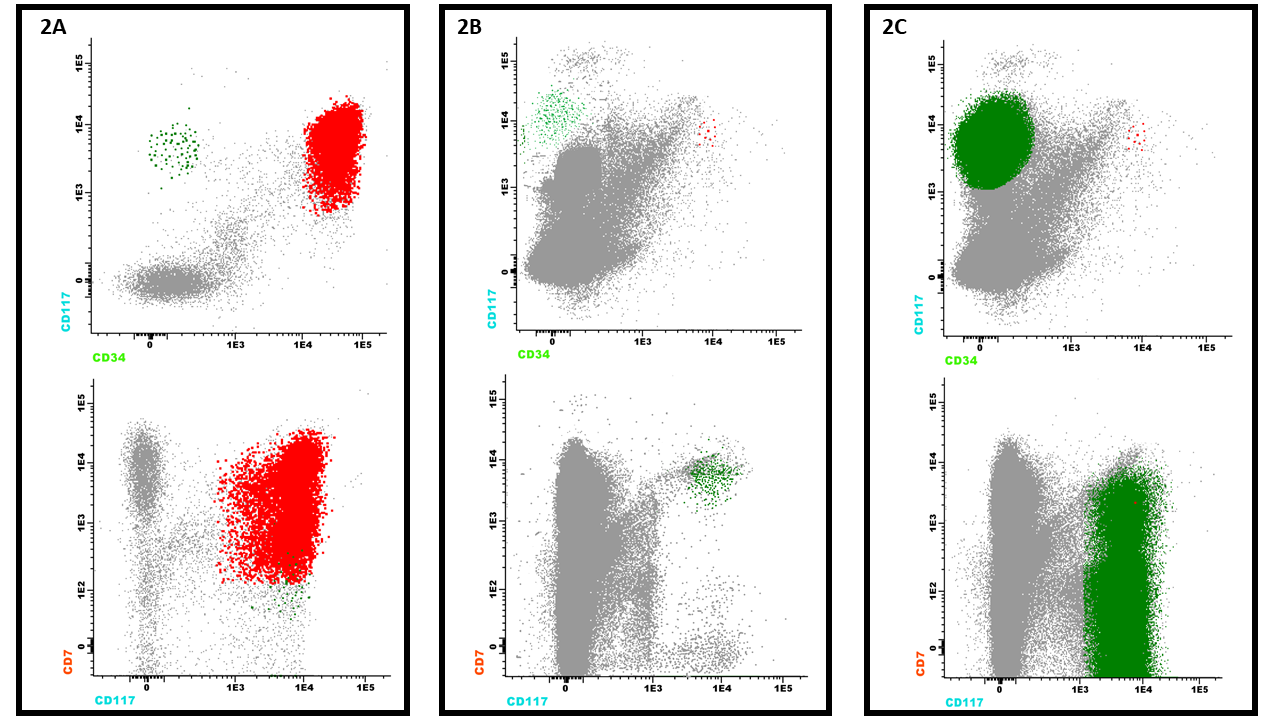
**Supplemental Figure 1. Visualization of standard gating strategy. (A)** FSC/SSC scatter plot for excluding debris. **(B)** Doublet discrimination by gating singlets (in yellow). **(C)** Selecting the WBC (in light blue) by gating the CD45 expressing population. **(D)** Gating immature blasts (dark blue) in the CD45dim/SSClow population. **(E)** Identifying lymphocytes (in green) which are high CD45 and low SSC. **(F)** Primitive/progenitor cells expressing CD34 within the blast population. **(G)** Final gate to select the LAIP (in this case CD13/CD56; in red) with an extra confirmation using the lymphocytes as reference. **(H)** Final confirmation by backgating of the LAIP cells on the CD45, CD34 and SSC/FSC plot. This can be used to ensure identification of appropriate population.

**Supplemental Figure 2. Example of upcoming clone in the Different from Normal (DfN) approach.** Concurrent leukemic populations may differently contribute to relapse in AML. **(A)** Flow cytometric immunophenotyping at diagnosis identified a predominant CD34+CD117+CD7+ clone (red) and a minority CD34-CD117+CD7+ sub-clone (green) that can be easily missed at first glance. **(B)** After induction chemotherapy, immunophenotyping showed an MRD negative status, with both population below the threshold of significance. **(C)** At relapse, the CD34+CD117+CD7+ population was still hardly detectable, whereas the CD34-CD117+CD7+ population had become the predominant leukemic clone. A closer look at empty spaces that ordinarily do not contain myeloid events has been crucial for the early identification of relapse.

**Supplemental Table S1. Most used Leukemia-associated immunophenotypes (LAIPs) in the consensus tube**

| Aberrant marker (combined with myeloid marker CD34 or CD117) | Reason for being a LAIP |
| --- | --- |
| CD117+/CD13- | CD13 should be on myeloid blasts |
| CD13-/CD33- | CD13 and CD33 should be on myeloid blasts, with follow-up pre-B-cells should be excluded |
| CD13+/CD33- | CD33 should be on myeloid blasts |
| CD45- | CD45 is a WBC marker |
| CD56+ | Is not present on normal blasts |
| CD7+ | Lymphocytic marker |
| HLA-DR- | HLA-DR should be positive for myeloid blasts |
| HLA-DR+/CD13- | CD13 should be on myeloid blasts |
| HLA-DR+/CD33- | CD33 should be on myeloid blasts |
| CD117+/CD33- | CD33 should be on myeloid blasts |
